# Identification of Transcription Factors and the Regulatory Genes Involved in Triacylglycerol Accumulation in the Unicellular Red Alga *Cyanidioschyzon merolae*

**DOI:** 10.3390/plants10050971

**Published:** 2021-05-13

**Authors:** Sota Takahashi, Riho Okubo, Yu Kanesaki, Baifeng Zhou, Kazuhiro Takaya, Satoru Watanabe, Kan Tanaka, Sousuke Imamura

**Affiliations:** 1 Interdisciplinary Graduate School of Science and Engineering, Tokyo Institute of Technology, Yokohama 226-8503, Japan; takahashi.s.bk@m.titech.ac.jp; 2 Laboratory for Chemistry and Life Science, Institute of Innovative Research, Tokyo Institute of Technology, Yokohama 226-8503, Japan; okubo.r.ab@m.titech.ac.jp (R.O.); zhou.b.aa@m.titech.ac.jp (B.Z.); kntanaka@res.titech.ac.jp (K.T.); 3 School of Life Science and Technology, Tokyo Institute of Technology, Yokohama 226-8503, Japan; 4 NODAI Genome Research Center, Tokyo University of Agriculture, Tokyo 156-8502, Japan; kanesaki.yuh@shizuoka.ac.jp; 5 Research Institute of Green Science and Technology, Shizuoka University, Shizuoka 422-8529, Japan; 6 NTT Space Environment and Energy Laboratories, Nippon Telegraph and Telephone Corporation, Tokyo 180-8585, Japan; kazuhiro.takaya.bm@hco.ntt.co.jp; 7 Department of Bioscience, Tokyo University of Agriculture, Tokyo 156-8502, Japan; s3watana@nodai.ac.jp

**Keywords:** algal biofuel, lysophosphatidic acid acyltransferase, red alga, transcription factor, triacylglycerol

## Abstract

Microalgal triacylglycerols (TAGs) are a good feedstock for liquid biofuel production. Improving the expression and/or function of transcription factors (TFs) involved in TAG accumulation may increase TAG content; however, information on microalgae is still lacking. In this study, 14 TFs in the unicellular red alga *Cyanidioschyzon merolae* were identified as candidate TFs regulating TAG accumulation using available transcriptome and phosphoproteome data under conditions driving TAG accumulation. To investigate the roles of these TFs, we constructed TF-overexpression strains and analyzed lipid droplet (LD) formation and TAG contents in the cells grown under standard conditions. Based on the results, we identified four TFs involved in LD and TAG accumulation. RNA-Seq analyses were performed to identify genes regulated by the four TFs using each overexpression strain. Among the TAG biosynthesis-related genes, only the gene encoding the endoplasmic reticulum-localized lysophosphatidic acid acyltransferase 1 (LPAT1) was notably increased among the overexpression strains. In the LPAT1 overexpression strain, TAG accumulation was significantly increased compared with the control strain under normal growth conditions. These results indicate that the four TFs positively regulate TAG accumulation by changing their target gene expression in *C. merolae*.

## 1. Introduction

Microalgae store energy in the form of storage molecules, such as neutral lipids that form cytoplasmic lipid droplets (LDs). The accumulated lipid is mainly in the form of triacylglycerols (TAGs), which can be harnessed for biofuel production, and the carbons of TAGs originate from the fixed CO_2_ via photosynthesis. Therefore, algal-based biofuels represent an important potential renewable energy system that could contribute to solving global warming [1,2,3]. However, large-scale industrial biofuel production systems using algae have not been constructed. A major reason for this is a lack of understanding of the underlying molecular mechanisms that control TAG accumulation in microalgae.

In our previous work, we had sought to clarify the mechanisms that regulate TAG biosynthesis using the unicellular model red alga *Cyanidioschyzon merolae* [4]. As observed with most microalgae, TAG content in these cells was enhanced after exposure to nitrogen depletion (–N) conditions [5]. Furthermore, we have elucidated that the TOR signaling pathway governs the accumulation of TAGs under –N conditions. TOR is a highly conserved protein kinase among eukaryotes, and it plays central roles in cell growth and stress responses by sensing environmental conditions, including available nutrients [6]. TOR kinase activity can be specifically inhibited by the TOR inhibitor rapamycin [7]. When we added rapamycin to the rapamycin-susceptible *C. merolae* F12 or SF12 strains [8,9] grown under standard growth conditions, the TAG content was increased to almost the same levels observed under –N conditions [5,9]. TAG accumulation after TOR inactivation was also observed for *Chlamydomonas reinhardtii* [10], *Euglena gracilis* [11], and *Phaeodactylum tricornutum* [12], indicating that the function of the TOR signaling pathway in modulating TAG accumulation is conserved among divergent eukaryotic algae [13].

Several studies have utilized enhanced expression of genes involved in TAG biosynthesis as a method for increasing TAG accumulation [14,15]. Driving the overexpression of transcription factors (TFs) that control the expression of TAG biosynthesis genes represents an efficient method for enhancing TAG accumulation, as each TF generally regulates sets of genes whose functions are interconnected [16]. Only a few TFs that control the accumulation of TAGs have so far been identified. For example, Boyle et al. identified NRR1, which encodes a SQUAMOSA promoter-binding protein domain TF, as induced under –N conditions. NRR1 is considered an important regulator of TAG synthesis under –N because the *nrr1* mutant produces only ~50% TAG compared with the wild type [17]. Goncalves et al. revealed that ROC40, a MYB-related TF, also has a role in –N-induced TAG accumulation, as the *roc40* mutant was impaired in its ability to increase the accumulation of TAG after exposure to –N [18].

In the present study, we used available transcriptome and phosphoproteome data obtained under –N and/or TOR inactivation conditions and identified four TFs and their regulatory genes that are involved in TAG accumulation in *C. merolae*. Based on these data, we demonstrate that one of the rate-limiting steps for TAG accumulation in this alga is catalyzed by endoplasmic reticulum (ER)-localized lysophosphatidic acid acyltransferase, which is regulated by the TFs.

## 2. Results

### 2.1. Identification of Candidate TFs Involved in TAG Accumulation

**Figure 1:**
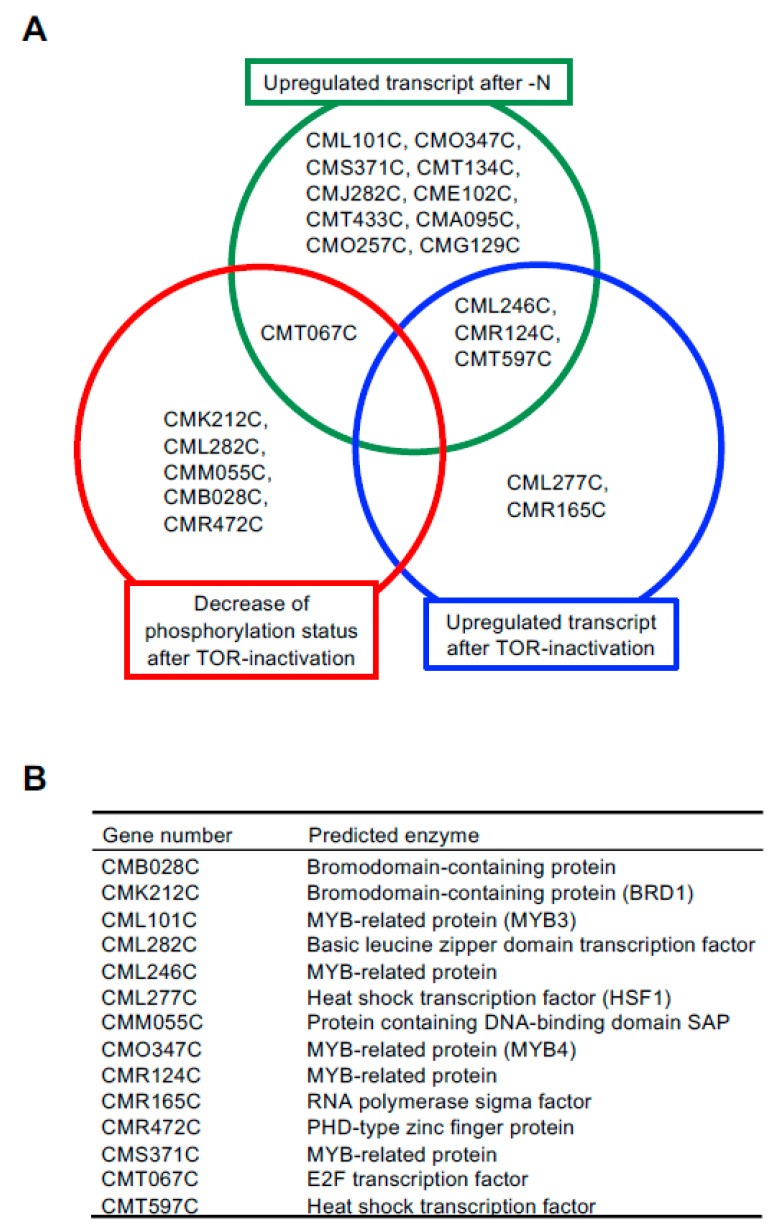
Identification of candidate TFs involved in TAG accumulation. (**A**) Venn diagram of the overlapping genes/proteins among the three datasets. (**B**) A list of the 14 proteins and their annotations analyzed in this study. Each gene number and annotation is assigned in the *C. merolae* database, http://czon.jp, accessed on 5 May 2021.

We previously revealed that TAG accumulation is accelerated under nitrogen depletion (–N) and TOR inactivation conditions in *C. merolae* [5,10]. To identify TFs with roles in TAG accumulation in *C. merolae* in this study, we first analyzed previously published microarray data [5] and phosphoproteome data [19] because it is generally observed that TFs exhibit changes in gene expression and/or post-translational modifications under environmental conditions where the TF is required [16]. Fourteen TFs (CML101C, CMO347C, CMS371C, CMT134C, CMJ282C, CME102C, CMT433C, CMA095C, CMO257C, CMG129C, CML246C, CMR124C, CMT597C, and CMT067C; each is a gene number in the *C. merolae* database, http://czon.jp, accessed on 5 May 2021) whose transcripts were increased under –N conditions and five TFs (CML277C, CMR165C, CML246C, CMR124C, and CMT597C) whose transcripts were increased after TOR inactivation by rapamycin were identified. In addition to these TFs, six TFs (CMK212C, CML282C, CMM055C, CMB028C, CMR472C, and CMT067C) whose phosphorylation status was decreased after TOR inactivation by rapamycin were identified. Among these TFs, four TFs (CML246C, CMR124C, CMT597C, and CMT067C) were identified in two out of three conditions (Figure 1B).

Before analyzing the mechanism by which these TFs influence TAG accumulation, we narrowed down the list of candidates from 10 TFs that were only upregulated by –N conditions. It was previously reported that ROC40, a MYB-related TF, has a role in –N-induced lipid accumulation, including TAG, in *Chlamydomonas* [18]. Among the 10 candidate TFs, a BLAST analysis revealed that CML101C, CMO347C, and CMS371C were homologs of ROC40, suggesting involvement of this TF in TAG accumulation in *C. merolae* as well. It is important to note that *C. merolae* possesses no NRR1 homolog [17]. Thus, in this study, we decided to analyze a total of 14 TFs (CMB028C, CMK212C, CML101C, CML282C, CML246C, CML277C, CMM055C, CMO347C, CMR124C, CMR165C, CMR472C, CMS371C, CMT067C, and CMT597C) (Figure 1B) for their possible regulation of TAG accumulation.

### 2.2. Effect of TF Overexpression on Lipid Droplet Formation and TAG Accumulation

If the 14 candidate TFs play critical roles in TAG accumulation in *C. merolae*, overexpression of each TF would potentially lead to an accumulation of cytoplasmic LDs that contain TAGs. To examine this possibility, we constructed overexpression strains for each candidate TF: CMB028Cox (CMB028C overexpression), CMK212Cox, CML101Cox, CML282Cox, CML246Cox, CML277Cox, CMM055Cox, CMO347Cox, CMR124Cox, CMR165Cox, CMR472Cox, CMS371Cox, and CMT067Cox. For the construction of overexpression strains, each TF gene was cloned into a pSUGA plasmid, which harbors UMP synthase. Expression of the TFs was regulated by the strong and constitutive *APCC* promoter [20]. The resultant plasmids were introduced into the *C. merolae* T1 strain [21], which lacks a UMP synthase and thus shows an uracil heterotrophic phenotype. The overexpression strains were selected on plates without uracil and confirmed by PCR with primers that can amplify exogenous plasmids containing each target gene (Figure S1). The TFc strain, into which the empty plasmid vector was introduced, was used as the control. As representative examples, we confirmed that significantly higher levels of *CMK212C*, *CML101C*, *CML277*, and *CMO347C* transcripts were present in each overexpression strain compared with TFc (Figure S2A). Furthermore, FLAG-fused TF proteins were detected (Figure S2B), confirming the successful construction of each overexpression strain. It should be noted that the overexpression strain for CMT597C has not yet successfully been constructed. While the reason is unknown, it is conceivable that increased expression of CMT597C protein may result in cell lethality. Thus, we excluded CMT597C from further analysis in this study.

**Figure 2:**
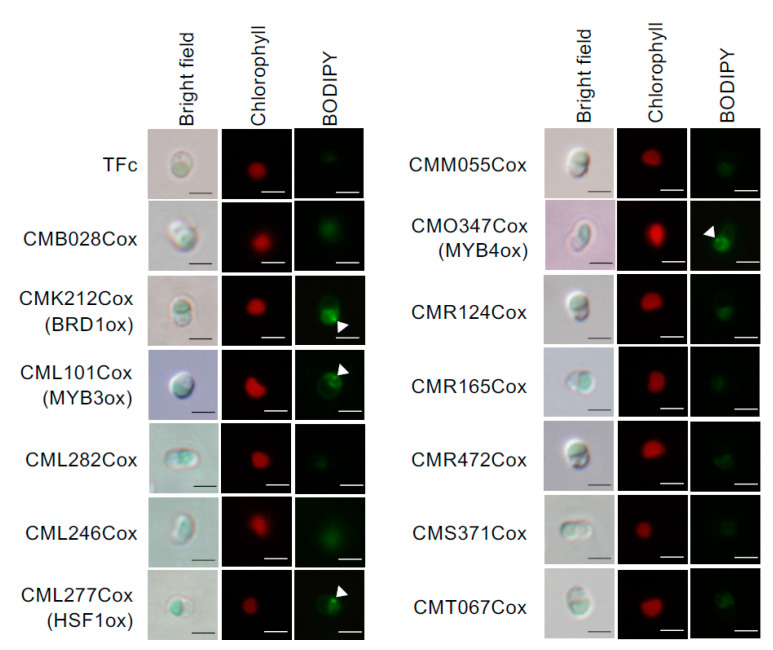
Accumulation of lipid droplets by overexpression of each TF. Overexpression strains were grown under normal growth conditions until OD_750_ = 0.4–0.6, and were stained with BODIPY. Bright field (**left**), chlorophyll fluorescence (center, red signal), and BODIPY staining (**right**, green signal) images are indicated with relevant strain names. TFc is the control strain for each overexpressor. Scale bar: 2 μm.

**Figure 3:**
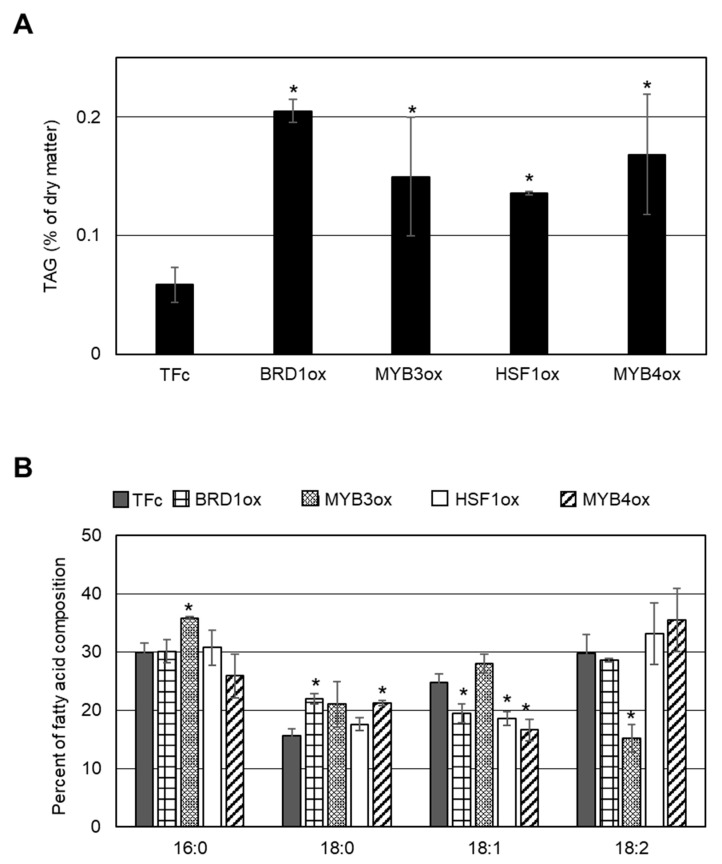
Accumulation of TAGs induced by BRD1, MYB3, HSF1, or MYB4 overexpression. (**A**) Intracellular TAG content in BRD1ox, MYB3ox, HSF1ox, MYB4ox, and TFc cells. Values are averages of three independent experiments and represent percentages of dry matter. Error bars indicate standard deviation (SD). Asterisks indicate a significant difference compared with TFc (Student’s *t*-test, *p* < 0.05). (**B**) Fatty acid composition of the purified TAGs in BRD1ox, MYB3ox, HSF1ox, MYB4ox, and TFc cells. Fatty acid components of the TAGs are indicated as a percentage of the fatty acid composition. Each fatty acid is indicated by the carbons:number of double bonds ratio. Values are averages of three independent experiments. Error bars indicate SD. Asterisks indicate a significant difference compared with TFc (Student’s *t*-test, *p* < 0.05).

We examined whether LD formation was induced in these overexpression strains under normal growth conditions. In BODIPY staining analyses, obvious LD formation was observed in CMK212Cox, CML101Cox, CML277Cox, and CMO347Cox, but not in the other overexpression strains and the control strain, TFc (Figure 2). We named CMK212C, CML101C, CML277C, and CMO347C as BRD1, MYB3, HSF1, and MYB4, respectively, and decided to analyze the four TFs hereafter (Figure 1B). We next measured TAG content using gas chromatography in the four overexpression strains that exhibited LD formation. Consistent with the BODIPY staining analysis, the TAG contents in BRD1ox (CMK212Cox), MYB3ox (CML101Cox), HSF1ox (CML277Cox), and MYB4ox (CMO347Cox) were significantly increased 2.2-, 3.2-, 3.8-, and 2.7-fold compared with the levels in TFc, respectively (Figure 3A). These results indicated that these four TFs are positively involved in TAG accumulation in *C. merolae*.

As shown in Figure 3B, the relative compositions of fatty acids in the TAGs were altered in each overexpressor compared with the TFc control. Comparing the carbons:number of double bonds ratio in fatty acids, we observed an increase of 16:0 and a decrease of 18:2 in MYB3ox; an increase of 18:0 and a decrease of 18:1 in MYB4ox; a decrease of 18:1 in HSF1ox; and an increase of 18:0 and a decrease of 18:1 in BRD1ox.

### 2.3. Comparison of TAG Synthesis-Related Gene Expression in BRD1ox, MYB3ox, HSF1ox, and MYB4ox

We postulated that the increases in LD formation and TAG accumulation in the four TF-overexpression strains would be driven by changes in the expression of TAG synthesis-related genes. Thus, we performed RNA-Seq analysis using RNAs isolated from the four overexpression strains and the control strain grown under the conditions shown in Figure 2 and Figure 3. As shown in Table 1, only CMJ021C, encoding lysophosphatidic acid acyltransferase (LPAT, we named it LPAT1), exhibited increased expression in all four overexpression strains, being 39.9.-fold in BRD1ox, 2.0-fold in MYB3ox, 133.5-fold in HSF1ox, and 13.0-fold in MYB4ox compared with TFc. This raised the possibility that the TAG accumulation observed in all four overexpression strains was due to an increase in LPAT1 expression.

### 2.4. TAG Accumulation in LPAT1 Overexpression Strain

To examine the role of *C. merolae* LPAT1 in TAG synthesis and its intracellular localization in the cells, we constructed a FLAG-fused LPAT1 overexpression strain, named LPAT1ox. The TFc strain was used as the control (see also Section 2.2). Quantitative real-time PCR (qRT-PCR) analysis revealed that *LPAT1* transcripts increased approximately 520-fold in LPAT1ox compared with TFc (Figure S3A). Furthermore, the FLAG-fused LPAT1 protein was detected at its predicted molecular weight in the strains using immunoblot analysis, indicating the successful construction of the LPAT1 overexpression strain (Figure S3B).

**Figure 4:**
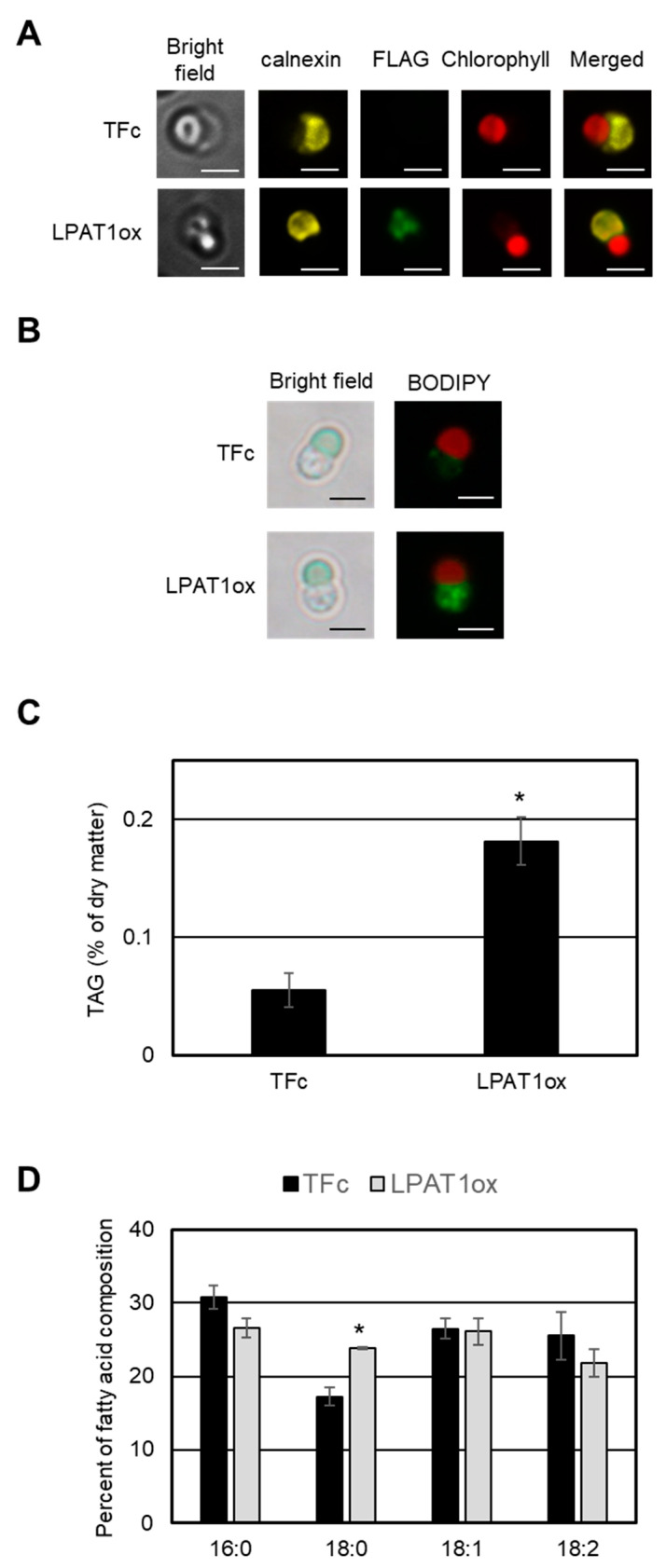
Accumulation of TAGs by LPAT1 overexpression. (**A**) Intracellular localization of FLAG-fused LPAT1. Fluorescence derived from FLAG-fused protein (yellow–green signal, FLAG), calnexin (yellow signal, calnexin), intrinsic chlorophyll fluorescence (red signal, Chlorophyll), merged image of FLAG and Chlorophyll (Merged), and bright field image (Bright field) in indicated strains are shown. Scale bars correspond to 2 μm. (**B**) BODIPY staining of LPAT1ox and TFc cells. Each cell was grown under normal growth conditions until OD_750_ = 0.4–0.6 and was stained with BODIPY. Bright field (left) and BODIPY staining (right) images are indicated. Each BODIPY staining (yellow–green signal) image was merged with the relevant chlorophyll fluorescence (red signal) image. Bar: 2 μm. (**C**) Intracellular TAG content in TFc and LPAT1ox cells. Values are averages of three independent experiments and represent percentages of dry matter. Error bars indicate SD. Asterisks indicate a significant difference compared with TFc (Student’s *t*-test, *p* < 0.05). The TAG contents were measured in two independent overexpression strains. The patterns are similar and we present data from one overexpression strain as a representative. (**D**) Fatty acid composition of the purified TAGs in LPAT1ox and TFc cells. Fatty acid components of the TAGs are indicated as a percentage of the fatty acid composition. Each fatty acid is indicated by the carbons:number of double bonds ratio. Values are averages of three independent experiments. Error bars indicate SD. Asterisks indicate a significant difference compared with TFc (Student’s *t*-test, * *p* < 0.05).

Using the FLAG epitope tag as a marker, we investigated the intracellular localization of LPAT1 via indirect immuno-fluorescence microscopy analysis. Yellow–green fluorescence showing the LPAT1 signal was observed in the cytosol, and the signal co-localized with a yellow fluorescence derived from ER-localized calnexin [22] (Figure 4A). This indirect immunofluorescence microscopy analysis indicated that LPAT1 is an ER-localized LPAT in *C. merolae*, and supports the hypothesis that LPAT1 is involved in the TAG synthesis.

If LPAT1 plays a critical role in TAG synthesis in *C. merolae*, the overexpression of LPAT1 would lead to accumulation of cytoplasmic LDs containing TAG. We thus first examined whether LD formation was induced in the LPAT1ox strain under normal growth conditions. In BODIPY staining analyses, obvious LD formation was observed in LPAT1ox but not in the TFc strain (Figure 4B). Consistent with the LD accumulation, the TAG content in LPAT1ox was significantly increased by 3.3-fold compared with the levels in TFc (Figure 4C). These data indicate that catalysis mediated by LPAT1 is an important step in TAG synthesis in *C. merolae.* As shown in Figure 4D, the relative cellular contents of 18:0 fatty acids in the TAGs in LPAT1ox were increased in comparison with those in TFc. This suggests that LPAT1 had a substrate preference for 18:0. 

## 3. Discussion

**Figure 5:**
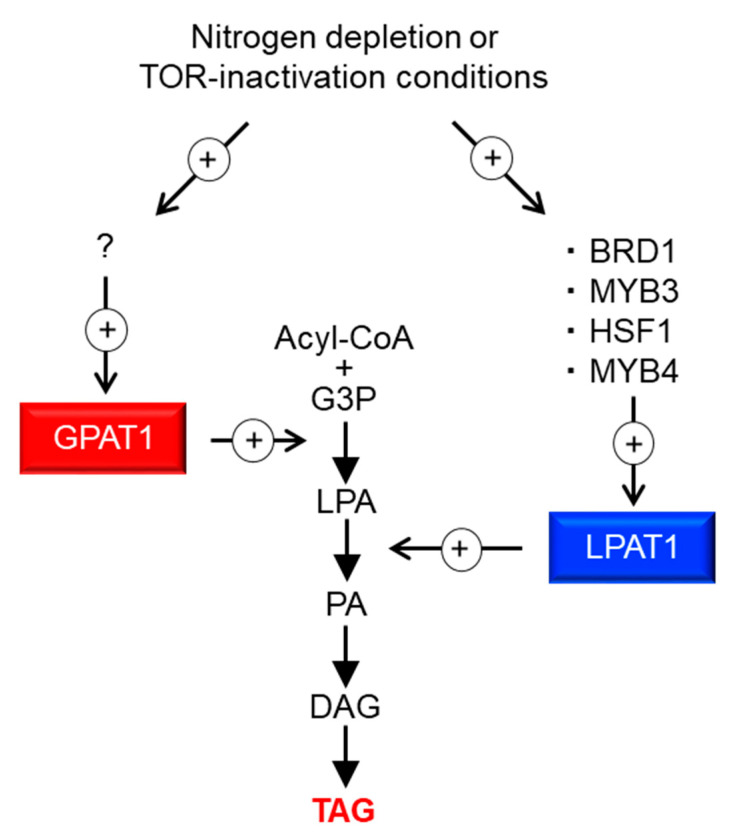
Possible model for the regulation of TAG synthesis in *C. merolae*. + with circle denotes positive effects. ? denotes an unidentified TF that regulates *GPAT1* transcripts. G3P, glycerol-3-phosphate; LPA, lysophosphatidic acid; PA, phosphatidic acid; DAG, diacylglycerol; TAG, triacylglycerol.

In this study, we identified four TFs involved in TAG accumulation; via the analysis of their target genes, we demonstrated that overexpression of ER-localized LPAT1 leads to TAG accumulation under normal growth conditions in *C. merolae*. Figure 5 illustrates a potential pathway for TAG accumulation through LPAT1. Under the –N or TOR inactivation conditions, the expression of four TFs (BRD1, HSF1, MYB3, and MYB4) increases, upregulating LPAT1 expression, and finally the resultant LPAT1 leads to TAG accumulation. TFs generally regulate sets of genes with interconnected functions, and thus, the identification of TFs involved in TAG accumulation is a valuable strategy for increasing TAG content. In this paper, we demonstrate the practicality and usefulness of this approach and elucidate fundamental knowledge regarding the regulation of TAG synthesis in microalgae.

Of the four identified TFs, MYB3 and MYB4 are single MYB-type TFs, signifying that they possess only one MYB domain that is involved in DNA-binding. It has been previously reported that ROC40, a single MYB-type TF, contributes to –N-induced lipid accumulation, including TAG, in *Chlamydomonas* [18]. Together, these findings suggest that single MYB-type TFs are involved in TAG accumulation in microalgae. In addition to the MYB-type TFs, two different types of TFs were identified as positive regulators of TAG accumulation in this study. HSF1 is annotated as a heat-shock TF in the *C. merolae* database (http://czon.jp, accessed on 5 May 2021). However, no induction of the *HSF1* transcript could be confirmed by microarray analysis using RNA isolated after a 1 h 50 °C treatment (Kanesaki et al., unpublished data). It is conceivable that HSF1 is a stress-responsive TF that is regulated depending on environmental conditions, such as nutritional deficiency, since HSF1 transcripts were induced under –N and TOR inactivation conditions by rapamycin [5]. The other TF we identified is BRD1, which is annotated as similar to bromodomain-containing TF in the database. Our previous study revealed that BRD1 is phosphorylated and its phosphorylation status is reduced after TOR inhibition by rapamycin, indicating that it is regulated by the TOR signaling pathway [19]. This suggests that BRD1 functions in response to changes in the external environment. The bromodomain is a domain that recognizes acetylated histones [23]; hence, states of histone modification could influence the expression of the gene targeted by BRD1, including *C. merolae* LPAT1. However, this process is unclear and should be addressed in future studies. Furthermore, examining the generality of the function of HSF1 and BRD1 homologs on TAG accumulation in other microalgae will elucidate fundamental TAG regulation mechanisms in microalgae.

The TAG fatty acid composition varied among the four TF-overexpressing strains (Figure 3B). This finding indicates that the functions of these TFs are similar (i.e., positively impacting TAG accumulation and LPAT1 expression) but not identical. In fact, the four identified TFs were not upregulated in common under –N and TOR inactivation conditions (Figure 1A). Therefore, it is possible to identify new genes regulated by each TF involved in TAG biosynthesis with the purpose of accumulating high amounts of TAG by upregulating the genes’ functions. These points are the next objectives to be investigated regarding the control of TAG biosynthesis and accumulation. This step-by-step approach based on the fundamental molecular regulation of TAG synthesis should lead to successful biofuel production using microalgae.

In this study, we demonstrated that the reaction catalyzed by LPAT1 is one of the rate-limiting steps in TAG synthesis in *C. merolae*. Recently, Muñoz et al. [24] investigated the ER-localized LPAT function of *Acutodesmus obliquus* in *Neochloris oleoabundans* cells. The authors showed that TAGs were significantly accumulated compared with the wild type under nitrogen-deficient conditions. These observations indicate that this step, which is catalyzed by ER-localized LPAT, is a good target for increasing the TAG content in microalgae in general. Furthermore, our recent study on *C. merolae* revealed that ER-localized GPAT1, which encodes glycerol-3-phosphate acyltransferase, is the major rate-limiting step of TAG biosynthesis. We observed an increase in TAG contents in the GPAT1-overexpression strain to approximately 2.1% of the dry matter (0.2% of dry matter in the case of LPAT1ox, see Figure 4C), which is comparable to that observed after 72 h under –N (3.1% of dry matter), even under normal growth conditions [15]. Therefore, at least two rate-limiting steps with different magnitudes are involved in TAG synthesis in *C. merolae* (Figure 5). As such, inducing the overexpression of LPAT1 or TFs that regulate LPAT1 expression (BRD1, HSF1, MYB3, or MYB4) in addition to GPAT1 would be a good strategy to increase the amount of TAG accumulation. Moreover, TF(s) that regulate *GPAT1* transcripts are currently unknown (“?” in Figure 5), but another strategy to increase intracellular TAG content could be via the overexpression of GPAT1-regulating TF(s) and/or the other positive regulators identified in this study. By contrast, in some green algae, it is generally accepted that the rate-limiting step of TAG synthesis is catalyzed by ER-localized diacylglycerol acyltransferase (DGAT) [25,26,27]; thus, the rate-limiting step in TAG synthesis is complex and varies depending on the microalgal strain. For further improvement of TAG accumulation in microalga cells, we should revalidate the established theory for the regulation of TAG synthesis in microalgae.

## 4. Materials and Methods

### 4.1. Strain and Growth Conditions

*Cyanidioschyzon merolae* 10D wild-type and T1 strains [9] were grown at 40 °C under continuous white light (50 µmol photons m^−2^ s^−1^) in liquid MA2 medium [28] at pH 2.5 bubbled with air supplemented with 2% (*v/v*) CO_2_.

### 4.2. Construction of Overexpression Strains

The 14 genes encoding TFs and the *LPAT1* gene were amplified with the primer sets shown in Table S1, using *C. merolae* genomic DNA as a template. Each PCR-amplified gene was then cloned into *Sma*I-digested pSUGA [20] using an In-Fusion HD cloning kit (Clontech). The T1 strain, which lacks UMP synthase and thus shows an uracil heterotrophic phenotype, was transformed with each plasmid as previously described [19] to obtain each overexpression strain. Each strain was selected on an MA2 plate [29] without uracil and confirmed by PCR with each forward primer used for the gene cloning and O250_Ter_Seq_R (5′-CCCTATCCACCGCAGAAGTA-3′) as a reverse primer. Each cloned protein was fused with a FLAG-tag and the expression was regulated by the strong *APCC* promoter [20] (Figure S1A).

### 4.3. Immunoblot Analysis

Immunoblot analysis was essentially performed as described previously [30,31]. Mouse anti-FLAG (Sigma) antibody was used at a dilution of 1:5000 to detect FLAG-fused protein. An HRP-conjugated anti-mouse (Promega) antibody was used as a secondary antibody at a dilution of 1:5000. The signals were detected using ImmunoStar Zeta (FUJIFILM Wako Pure Chemical Industries) and a chemiluminescent image analyzer Lumino Graph I (WSE-6100H, ATTO CORPORATION, Tokyo, Japan).

### 4.4. Lipid Analysis

Staining and detection of LDs with BODIPY 505/515 (4,4-difluoro-1,3,5,7-tetramethyl-4-bora-3a,4a-diaza-s-indacene) and quantification of TAGs by gas chromatography were performed as described previously [5,15].

### 4.5. RNA Preparation

*C. merolae* cells in about 50 mL of culture were harvested at 1200× *g* for 10 min. The supernatant was discarded, the pellet was dissolved in 500 µL of RNA extraction buffer (50 mM Tris-HCl pH 6.8, 5 mM EDTA, 0.5% (*w/v*) SDS), and then 500 µL of acidic phenol was added. The tube was next incubated at 65 °C for 8 min, with intermittent mixing. After centrifugation at 18,000× *g* for 10 min, the supernatant was mixed with an equal volume of phenol:chloroform:isoamyl alcohol (25:24:1), the tube was centrifuged again, and the supernatant was collected in a new tube and ethanol-precipitated. Isolated RNA was treated with RQ1 RNase-Free DNase (Promega), and the reaction mixture was mixed with an equal volume of phenol:chloroform:isoamyl alcohol (25:24:1). After centrifugation (18,000× *g* for 10 min), the supernatant was collected in a new tube and ethanol-precipitated. Isolated RNA was subjected to qRT-PCR analysis and RNA-Seq analysis (as described below).

### 4.6. Quantitative Real-Time PCR Analysis

qRT-PCR analysis was performed as described [5] with some modifications. In the step of complementary DNA (cDNA) synthesis, cDNA fragments were synthesized using 500 ng total RNA with ReverTra Ace^®^ qPCR RT Master Mix with gDNA Remover Kit (TOYOBO), according to the manufacturer’s protocol. Primers used in qRT-PCR analyses are shown in Table S2.

### 4.7. RNA-Seq Analysis

Total RNA was extracted from *C. merolae* cells as described above. One microgram of total RNA was used for library synthesis as follows. Both nuclear and organelle ribosomal RNA was removed using the RiboZero rRNA Removal Kit (Plant Leaf) (Illumina) following the manufacturer’s protocol. Sequencing libraries were prepared using the NEBNext Ultra mRNA library prep kit for Illumina (NEB) with the following modifications. The random hexamer primer was used for reverse transcription. After second strand synthesis, double-stranded cDNA was fragmented to an average length of 300 bp using a Covaris S2 sonication system (Covaris, Woburn, USA). One hundred cycles of paired-end sequencing were carried out using the HiSeq2500 system according to the manufacturer’s specifications (Illumina, San Diego, USA). After the sequencing reactions were complete, the Illumina analysis pipeline (CASAVA 1.8.0) was used to process the raw sequencing data. The RNA-Seq reads were trimmed using CLC Genomics Workbench ver. 11.0. with the following parameters; Phred quality score >30; automatic removal of read-through adaptor: yes; removing terminal 15 nucleotides from the 5′ end and 2 nucleotides from the 3′ end; removing truncated reads less than 50 nucleotides in length. Trimmed reads were mapped to the all genes in the reference genome of *Cyanidioschyzon merolae* 10D (accession number: AP006483.2 for nucleus, NC_000887.3 for mitochondrion, and AB002583.1 for chloroplast) using CLC Genomics Workbench ver. 11.0. (Qiagen) with the following parameters; mismatch cost: 2; Indel cost: 3; length fraction: 0.55; similarity fraction: 0.9; global alignment: no; maximum number of hits for a read: 1. The numbers of raw data reads, trimmed data, and mapped data are listed in Table S3. The expression level of each gene was calculated by counting the uniquely mapped reads to each gene, and was normalized by calculating TPM values. The read 1 and read 2 data of paired-end reads were used separately for mapping analysis, and expression values were calculated by averaging of these results (*n* = 1). For comparison of the expression level of genes, pseudocounts were added to both numbers of reads to avoid division by zero. Original sequence data were deposited into the DDBJ Sequence Read Archive (DRA) with the accession numbers DRR258822–DRR258826. 

### 4.8. Indirect Immuno-Fluorescence Microscopy Analysis

The TFc and the LPAT1ox strains grown under the normal growth conditions were fixed with 1% (*w/v*) paraformaldehyde and 10% (*v/v*) dimethyl sulfoxide in methanol. Each fixed cell was reacted with a mouse anti-DYKDDDDK FLAG antibody (FUJIFILM Wako Pure Chemical Industries) and a rat anti-calnexin antibody at the same time in 5% (*v/v*) blocking one (nacalai tesque) in PBS. The localizations of LPAT1 and calnexin were detected with Alexa Fluor 488-conjugated goat anti-mouse IgG antibody and Alexa Fluor 555-conjugated goat anti-rat IgG antibody (Thermo Fisher Scientific), respectively. The fluorescence derived from the secondary antibody and chlorophyll was observed by fluorescence microscopy (BX51, Olympus, Tokyo, Japan).

## Figures and Tables

**Table 1:** A list of the predicted fatty acid- and TAG synthesis-related genes and their expression ratios in BRD1ox, MYB3ox, HSF1ox and MYB4ox strains.

Gene Number ¹	Predicted Enzyme	Ratio (vs. TFc)			
		BRD1ox (CMK212Cox)	MYB3ox (CML101Cox)	HSF 1ox (CML277Cox)	MYB4ox (CMO347Cox)
CMS299C	Biotin carboxylase, chloroplast precursor	0.9	1.2	1.5	1.2
CMT420C	Malonyl-CoA ACP transacylase	1.2	1.5	1.4	2.0
CMM286C	3-ketoacyl-ACP synthase	1.9	2.2	2.4	1.9
CML329C	3-ketoacyl-ACP synthase	1.0	1.0	1.0	1.0
CMD118C	3-ketoacyl-ACP synthase	0.9	1.4	1.1	1.5
CMS393C	3-keotacyl-ACP reductase	1.0	1.4	1.5	1.7
CMI240C	3-hydroxyacyll-ACP dehydratase	0.6	1.0	0.8	1.2
CMT381C	Enoyl-ACP reductase	1.3	1.5	1.6	1.6
CMJ027C	Glycerol-3-phosphate acyltransferase	1.1	1.5	1.9	2.1
CMA017C	Glycerol-3-phosphate acyltransferase	1.0	1.4	1.4	1.8
CMK217C	Glycerol-3-phosphate acyltransferase	1.1	1.2	1.3	2.1
CME109C	Lysophosphatidic acid acyltransferase	1.0	1.3	1.4	1.9
CMF185C	Lysophosphatidic acid acyltransferase	0.9	1.0	1.2	1.8
CMJ021C	Lysophosphatidic acid acyltransferase (LPAT1)	39.9	2.0	133.5	13.0
CMR054C	Phosphatidic acid phosphatase	0.8	1.2	1.7	1.6
CMR488C	Phosphatidic acid phosphatase	1.5	1.6	2.3	1.8
CMQ199C	Diacylglycerol acyltransferase	1.0	1.2	1.3	1.9
CME100C	Diacylglycerol acyltransferase	0.7	0.8	0.8	1.4
CMJ162C	Diacylglycerol acyltransferase	1.1	1.4	1.5	2.1
CMB069C	Diacylglycerol acyltransferase	1.1	1.3	2.4	1.9

^1^ Gene number in *C. merolae* database, http://czon.jp accessed on 5 May 2021.

## Supplementary Materials

The following are available online at https://www.mdpi.com/article/10.3390/plants10050971/s1, Figure S1: Construction of the transcription factor (TF)-overexpression strain; Figure S2: Overexpression of BRD1, MYB3, HSF1, and MYB4; Figure S3: Overexpression of LPAT1.
