# Supplementary material for: Identification of Transcription Factors and the Regulatory Genes Involved in Triacylglycerol Accumulation in the Unicellular Red Alga Cyanidioschyzon merolae
Source: Plants (Basel). 2021 May 13;10(5):971. doi: 10.3390/plants10050971 (PMC8152781; doi:10.3390/plants10050971)
Supplement: Supplementary file 1 [file plants-10-00971-s001.zip › Table S1.pdf]

Table S1 Primers used for construction of overexpression strains.

Gene	Primer	Sequence (5′-3′)
CMT067C	T067_Fw	TTCTTCGTTCGTTGACCCCCATGAGCGAATCTGCTGCCGC
	T067_Rv	TGCAGGTCGACTCTAGACCCGCTCAGCGAGCGTTCAAAAT
CML246C	L246_Fw	TTCTTCGTTCGTTGACCCCCATGGAGGCGACACTGCGGCG
	L246_Rv	TGCAGGTCGACTCTAGACCCTAGCATATTCTCCTGTGCAA
CMR124C	R124_Fw	TTCTTCGTTCGTTGACCCCCATGGATATTTCTGGCAGTGC
	R124_Rv	TGCAGGTCGACTCTAGACCCTGATCTGTCCAGGTCTTCAG
CMT597C	T597_Fw	TTCTTCGTTCGTTGACCCCCATGGACTTATTGGCAAAAGC
	T597_Rv	TGCAGGTCGACTCTAGACCCCTTGCCTGGTAGCGGTACAT
CMR165C	R165_Fw	TTCTTCGTTCGTTGACCCCCATGTTCACGCAGGGACGTAC
	R165_Rv	TGCAGGTCGACTCTAGACCCCAGTGCGCTTGTGTTCAGCG
CML277C	L277_Fw	TTCTTCGTTCGTTGACCCCCATGACAGAGAGTGTAACCAA
(HSF1)	L277_Rv	TGCAGGTCGACTCTAGACCCGTCTTGCTCTGGTGTATCTT
CMO347C	O347_Fw	TTCTTCGTTCGTTGACCCCCATGGTGCAGGACCCGGTAGT
(MYB4)	O347_Rv	TGCAGGTCGACTCTAGACCCGGTTGCAGCGAGGAGAGCGG
CMS371C	S371_Fw	TTCTTCGTTCGTTGACCCCCATGGGGTCCGAAAAGCATGA
	S371_Rv	TGCAGGTCGACTCTAGACCCCGAATAGGGCATGCTCTCTT
CML101C	L101_Fw	TTCTTCGTTCGTTGACCCCCATGGACCCAGGCAAGTCCAC
(MYB3)	L101_Rv	TGCAGGTCGACTCTAGACCCGGGAAACGGCAGCGACGATG
CMK212C	K212_Fw	TTCTTCGTTCGTTGACCCCCATGGAAGACGGGGCACAGGG
(BRD1)	K212_Rv	TGCAGGTCGACTCTAGACCCGTATTCGTCCCCATCGCTGG
CML282C	L282_Fw	TTCTTCGTTCGTTGACCCCCATGGAAGGAGACCTGCACGC
	L282_Rv	TGCAGGTCGACTCTAGACCCGAGCACGGACGCAGGCTCCT
CMM055C	M055_Fw	TTCTTCGTTCGTTGACCCCCATGAACGCTGCGGTTCTTCG
	M055_Rv	TGCAGGTCGACTCTAGACCCCTTTGAGGCGTTCGCAGCTT
CMB028C	B028_Fw	TTCTTCGTTCGTTGACCCCCATGCAGCGTGTCGATGGCGC
	B028_Rv	TGCAGGTCGACTCTAGACCCAACCTGCGGTCTGAGCATAG
CMR472C	R472_Fw	TTCTTCGTTCGTTGACCCCCATGAGCGAAGACTCACGCAC
	R472_Rv	TGCAGGTCGACTCTAGACCCTTTGGGGCGAGCTCGCACGT
CMJ021C	J021_Fw	TTCTTCGTTCGTTGACCCCCATGATCCGTGATCCATACCGA
(LPAT1)	J021_Rv	TGCAGGTCGACTCTAGACCCTGATCCGCAGTTTGGATCGCA
