# Supplementary material for: Identification of Transcription Factors and the Regulatory Genes Involved in Triacylglycerol Accumulation in the Unicellular Red Alga Cyanidioschyzon merolae
Source: Plants (Basel). 2021 May 13;10(5):971. doi: 10.3390/plants10050971 (PMC8152781; doi:10.3390/plants10050971)
Supplement: Supplementary file 1 [file plants-10-00971-s001.zip › Table S2.pdf]

Table S2 Primers used for qRT-PCR analysis.

Gene	Primer	Sequence (5′-3′)
CML277C	L277_QRT_F1	ATTGAGCTCATTCGCAACCT
(HSF1)	L277_QRT_R1	GAGGTGGAGACCACTGCAAC
CMO347C	O347_QRT_F1	TATCACTCCCACCCGACATT
(MYB4)	O347_QRT_R1	TCCCTCGCTTTCAAATTGTT
CML101C	L101_QRT_F1	TCTAGAGGAGGCATCGGAGA
(MYB3)	L101_QRT_R1	CTCCTCGGGTACCATGAAAA
CMK212C	K212_QRT_F1	GTCGAGCTATTCGGTTCTCG
(BRD1)	K212_QRT_R1	AGTCTCATTGCTGGCCTGTT
CMJ021C	J021_QRT_F1	TTTCTAGGCCGGTTTTTCAA
(LPAT1)	J021_QRT_R1	GTCGACTATCCCGCTCAATC
