# Supplementary material for: Identification of Transcription Factors and the Regulatory Genes Involved in Triacylglycerol Accumulation in the Unicellular Red Alga Cyanidioschyzon merolae
Source: Plants (Basel). 2021 May 13;10(5):971. doi: 10.3390/plants10050971 (PMC8152781; doi:10.3390/plants10050971)
Supplement: Supplementary file 1 [file plants-10-00971-s001.zip › Table S3.pdf]

Table S3. Sequence read counts of raw data, trimmed data, and mapped data.

Sample name	Total read pairs		Number of reads after quality trimming	Number of mapped reads
TFc	16,034,815	read1	14,461,313	13,578,559
		read2	14,547,270	13,659,335
BRD1ox (CMK212Cox)	14,836,994	read1	13,403,136	12,713,115
		read2	13,484,513	12,791,752
MYB3ox (CML101Cox)	15,230,883	read1	13,676,879	12,824,716
		read2	13,774,656	12,917,260
HSF1ox (CML277Cox)	9,868,974	read1	8,846,548	8,327,980
		read2	8,891,290	8,370,375
MYB4ox (CMO347Cox)	13,350,494	read1	11,940,064	11,155,619
		read2	12,000,539	11,211,358
